# HOSPICE VISIT INTENSITY BY PHYSICIANS AND NURSE PRACTITIONERS ON THE GENERAL INPATIENT LEVEL OF CARE

**DOI:** 10.1093/geroni/igz038.021

**Published:** 2019-11-08

**Authors:** Thomas J Christian, Joan Teno, Pedro L Gozalo, Michael Plotzke

**Affiliations:** 1 Abt Associates, Cambridge, Massachusetts, United States; 2 Oregon Health & Science University, Portland, Oregon, United States; 3 Brown University, Providence, Rhode Island, United States

## Abstract

The Medicare Hospice Benefit’s General Inpatient (GIP) level of care provides short-term services for pain and symptom management in an inpatient facility that cannot be managed in the patient’s home. Relatively little is known about how beneficiaries utilize services during GIP care. Among a cohort of Medicare hospice beneficiaries utilizing GIP during Federal Fiscal Year 2014 (FY2014), we used 100% Medicare hospice and Part B claims to identify physician and nurse practitioner services concurrent with GIP dates. We estimated logistic regression models to determine the likelihood a beneficiary never receives physician or nurse practitioner services. We found that among the 1.5 million GIP days serviced in FY2014, more than half (52.4%) lacked any recorded physician or nurse practitioner services. Absence rates for these services were particularly high among hospice GIP days provided in inpatient facilities (69.1% missing services), long-term care hospitals (84.3% missing services), and skilled nursing facilities (85.3% missing services). Moreover, one in five hospice episodes having at least three sequential GIP days lacked any physician or nurse practitioner services. Relative to hospice inpatient units, rates of absence were higher among episodes beginning in long-term care hospitals [59.3% long-term care hospital vs. 11.5% hospice inpatient units; AOR 9.65 95% CI 7.47-12.46] and skilled nursing facilities [51.3% skilled nursing facility vs. 11.5% hospice inpatient units; AOR 5.98, 95% CI 5.63-6.36]. More in depth research and monitoring is needed to further understand dimensions of GIP care provision, to ensure that hospice beneficiaries are receiving adequate services regardless of their inpatient setting.

